# Progression From Early Multiterritorial Punctate Infarcts to Massive Stroke in Fulminant Thrombotic Thrombocytopenic Purpura Despite Aggressive Therapy: A Therapeutic Dilemma in Initiating Antithrombotic Therapy

**DOI:** 10.7759/cureus.88250

**Published:** 2025-07-18

**Authors:** Zhuo Luan

**Affiliations:** 1 Neurology, Texas Tech University Health Sciences Center El Paso, EL Paso, USA

**Keywords:** adamts13 deficiency, middle cerebral artery infarction, stroke, thrombotic thrombocytopenic purpura, treatment choices

## Abstract

Thrombotic thrombocytopenic purpura (TTP) is a rare, life-threatening thrombotic microangiopathy characterized by a profound deficiency in ADAMTS13 activity. It can present with a wide range of clinical features, including thrombocytopenia, hemolytic anemia, renal impairment, fever, and neurologic manifestations. Prompt recognition and treatment with plasma exchange and immunosuppressive therapy significantly improve survival. However, in rare cases, TTP may become refractory to aggressive treatment and progress rapidly. We report the case of a 62-year-old woman who presented with abdominal pain and acute confusion and was diagnosed with TTP complicated by multiterritorial punctate cerebral infarcts, following laboratory findings that revealed severe thrombocytopenia, hemolytic anemia, and markedly reduced ADAMTS13 activity. Despite aggressive treatment with plasma exchange, corticosteroids, rituximab, cyclophosphamide, and caplacizumab, the patient’s condition worsened, progressing to a large left middle cerebral artery ischemic stroke in addition to multifocal infarctions, seizures, encephalopathy, and ultimately, respiratory failure. This case highlights the diagnostic and therapeutic challenges of refractory TTP and underscores the devastating neurologic complications that can occur despite maximal medical therapy. It also raises important questions about the dilemma of whether or when to initiate antithrombotic therapy in the setting of severe thrombocytopenia and ongoing microvascular thrombosis.

## Introduction

Thrombotic thrombocytopenic purpura (TTP) is a rare but potentially fatal hematologic emergency characterized by the formation of microvascular platelet-rich thrombi due to a severe deficiency of ADAMTS13 (a disintegrin and metalloproteinase with thrombospondin type 1 motif, member 13) [1]. This metalloprotease is critical for cleaving ultra-large multimers of von Willebrand factor (vWF), and its deficiency, most often acquired through autoantibodies, increases the risk of spontaneous platelet aggregation and widespread microvascular thrombosis [2]. Classically, TTP was defined by a pentad of clinical features: thrombocytopenia, microangiopathic hemolytic anemia, neurologic symptoms, renal dysfunction, and fever. However, most patients do not present with the full pentad, and diagnosis is often based on thrombocytopenia and hemolytic anemia in the appropriate clinical setting. Prompt recognition and treatment are essential, as delays in therapy are associated with poor outcomes [3]. Before the advent of plasma exchange (PLEX) in the 1980s, the mortality rate of TTP exceeded 90%. Today, with the use of PLEX, corticosteroids, rituximab, and, more recently, caplacizumab, survival rates have improved dramatically, with current estimates showing remission in over 80-90% of cases when appropriately treated [4].

Despite these advances, a subset of patients experience a fulminant or refractory course, marked by a lack of hematologic response and progressive end-organ damage, particularly in the central nervous system. Neurological complications are among the most common and devastating manifestations of TTP [5]. Symptoms can range from mild confusion and headache to seizures, encephalopathy, and ischemic strokes. In some cases, neurologic deterioration can occur rapidly, especially when complicated by stroke, even after therapy has been initiated, leading to long-term disability or death [6,7]. Ischemic stroke occurs in approximately 5-10% of acute immune-mediated TTP. Stroke types are often small and multifocal without a specific distribution pattern, though a few cases of large artery strokes have been reported [8].

Managing ischemic stroke in a patient with TTP presents a unique and critical challenge, particularly when antithrombotic therapies are contraindicated due to severe thrombocytopenia, precisely the time when they may be needed most to prevent stroke progression. According to the updated TTP treatment guidelines in 2025, antiplatelets are generally not recommended in TTP unless there is an ischemic stroke. They may be harmful in the acute phase of TTP when the platelet count is below 50×10⁹/L, based on current literature [9]. However, there is a lack of detailed studies on fulminant cases. The profound thrombocytopenia that characterizes TTP renders patients ineligible for standard ischemic stroke interventions such as antiplatelets, anticoagulants, or thrombolytics [10]. Thus, determining whether and when to initiate antithrombotic therapy remains a significant therapeutic dilemma in the management of TTP-associated stroke. This dilemma becomes even more pronounced in refractory or fulminant TTP, where disease-directed treatments alone, despite timely use, may not be sufficient to prevent stroke progression. To our knowledge, no prior case reports have documented the progression of ischemic stroke occurring in the setting of fulminant TTP despite comprehensive therapy.

In this report, we present a case of acquired TTP in a 62-year-old woman who developed progressive multifocal cerebral infarctions and ultimately a large territorial stroke, despite timely and comprehensive treatment with plasma exchange, corticosteroids, rituximab, cyclophosphamide, and caplacizumab. Antithrombotic therapy could not be initiated due to profound thrombocytopenia. This case highlights the need for further research to address the therapeutic challenge of initiating antithrombotic therapy in TTP, with the goal of preventing stroke and halting its progression.

## Case presentation

**Figure 1:**
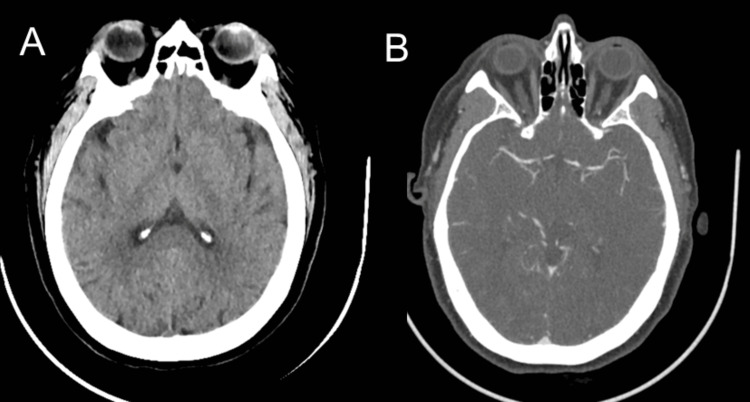
Initial CT and CTA of the head were normal. Computed tomography (CT) (A) and computed tomography angiography (CTA) of the head (B), showing no hemorrhage and no large vessel occlusion (LVO).

A 62-year-old woman with a history of hypertension presented with two days of abdominal pain and nausea, followed by confusion and speech difficulty. According to the family, she appeared disoriented and was unable to recognize familiar people or her surroundings. On arrival at the emergency department, a stroke code was activated. Her initial National Institutes of Health (NIH) Stroke Scale (NIHSS) score was two due to being unable to answer the month and her age correctly. The CT head and CT angiography of the head and neck were unremarkable, showing no hemorrhage or large vessel occlusion (Figure 1).

On physical examination, GCS was 13; the patient was alert but disoriented to time and place. Cranial nerve examination showed horizontal nystagmus but no facial weakness. Motor strength was preserved in all extremities. Laboratory evaluation revealed severe thrombocytopenia and anemia, along with elevated lactate dehydrogenase, undetectable haptoglobin, and increased total bilirubin. ADAMTS13 activity was undetectable, with a high inhibitor level, confirming the diagnosis of TTP (Table 1).

**Table 1:** Laboratory results from initial presentation and on day 10 after treatment. WBC: White blood cells, RBC: Red blood cells, HGB: Hemoglobin, PLT: Platelets, BUN: Blood urea nitrogen, AST: Aspartate aminotransferase, ALT: Alanine aminotransferase, ALK phos: Alkaline phosphatase, LDH: Lactate dehydrogenase Lab tests showed low platelets, anemia, high LDH, undetectable haptoglobin, high bilirubin, and very low ADAMTS13 activity with high inhibitor levels, confirming acquired TTP. Even after aggressive treatment, by day 10 the platelet count and ADAMTS13 activity were still low, and the inhibitor levels remained high, with only slight improvement.

Blood/Serum	Admission	Day 10	Reference
WBC	11.26	13.03	4.5-11 x10^3^/uL
RBC	2.15	2.38	3.5-5.5 x10^6^/uL
HGB	6.8	7.8	12-15 g/DL
PLT	8	21	150-450 x10^3^/uL
BUN	43	30	7-17 mg/dL
Creatinine	1.6	0.5	0.52-1.04 mg/dL
Total bilirubin	4.8	1.6	0.2-1.3 mg/dL
Direct bilirubin	1.6	-	0-0.3 mg/dL
AST	61	30	14-36 IU/L
ALT	36	36	0-35 IU/L
ALK phos	95	99	38-126 IU/L
LDH	1536	-	120-246 IU/L
Haptoglobin	<10	-	43-212 mg/dL
ADAMTS13 Activity	<0.03	0.11	0.68-1.63 IU/mL
ADAMTS13 Inhibitor	3.7	1.3	<0.4 BEU

**Figure 2:**
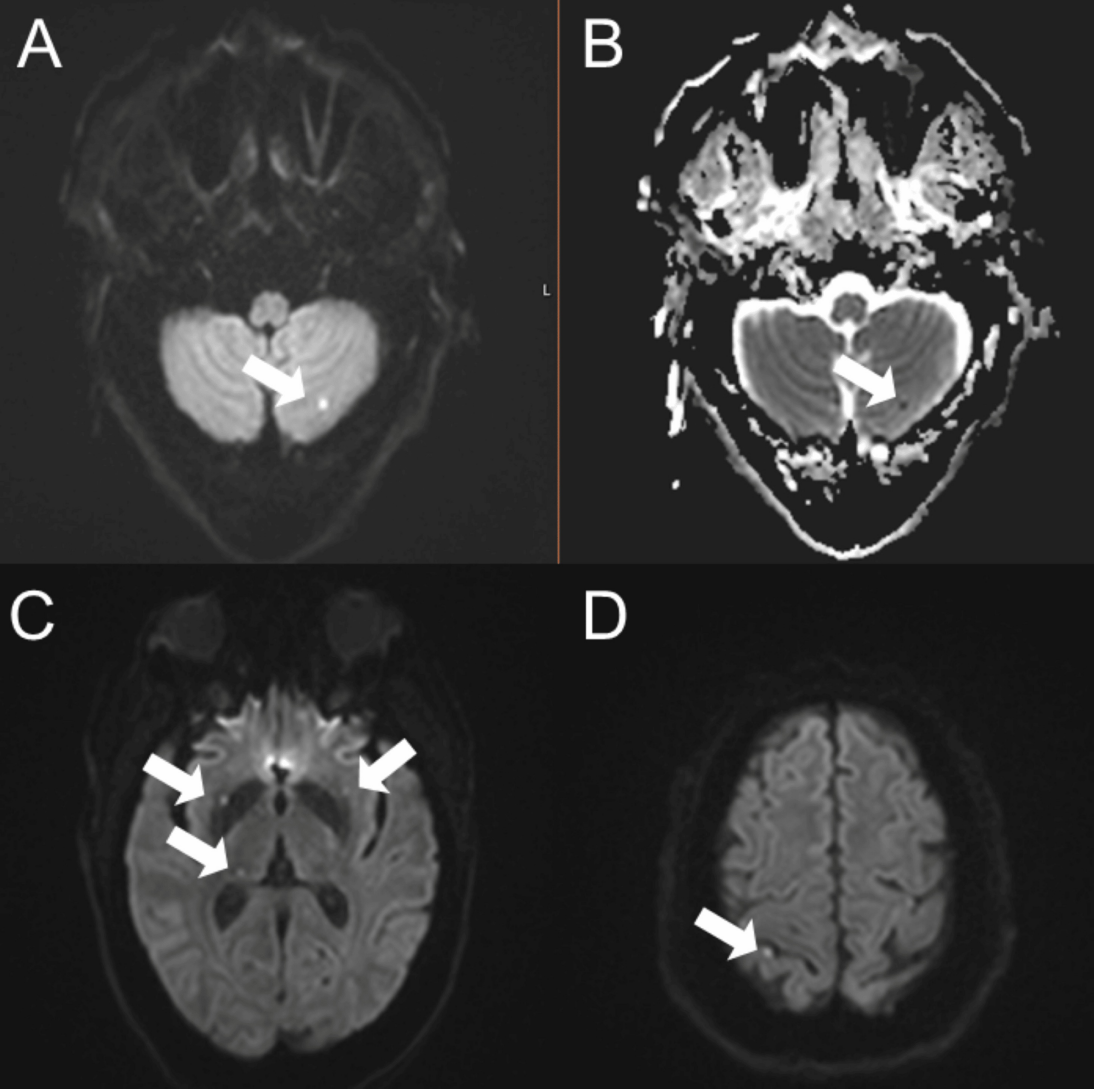
Brain MRI showed numerous acute punctate infarcts consistent with a multiterritorial ischemic stroke. Axial diffusion-weighted imaging (A) reveals focal diffusion restriction in the left cerebellum (arrows), confirmed by true restricted diffusion on the corresponding ADC map (B). Additional DWI images demonstrate infarcts in the basal ganglia and thalami (C), as well as the frontal lobes (D). ADC: Apparent diffusion coefficient, DWI: Diffusion-weighted imaging

The patient was admitted to the intensive care unit and began daily plasma exchange with transfusion as needed, along with high-dose intravenous methylprednisolone. Despite therapy, she developed a generalized tonic-clonic seizure on day three and was started on levetiracetam and lacosamide. The patient became obtunded following the seizures and was intubated for airway protection. Brain MRI revealed acute multiple punctate infarcts in bilateral basal ganglia, thalami, frontal lobes, and cerebellum (Figure 2). Rituximab was initiated on hospital day four. Over the next several days, her platelet count remained critically low, and additional immunosuppressive therapy with IV cyclophosphamide was initiated. Caplacizumab was also added.

**Figure 3:**
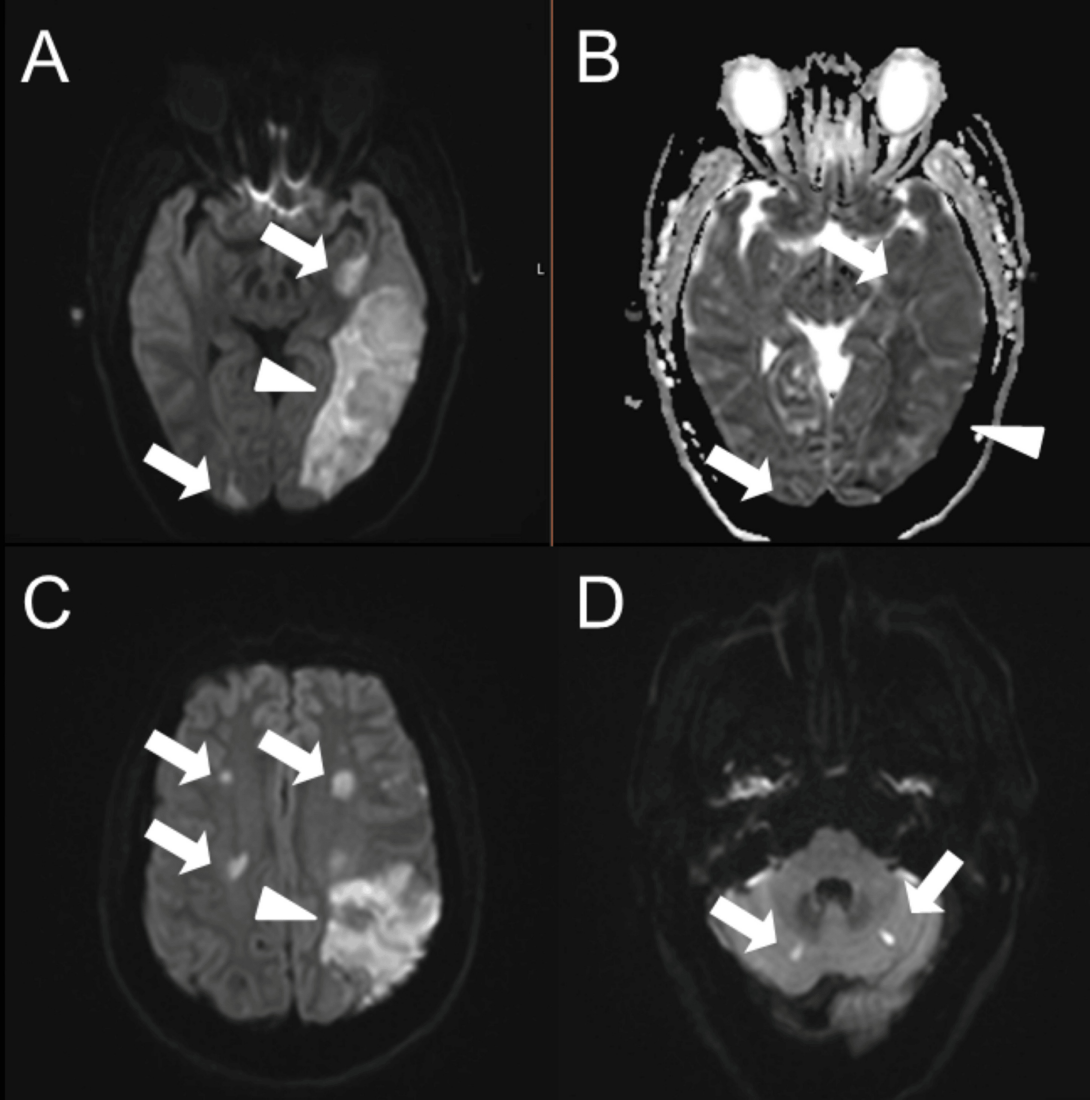
Follow-up brain MRI demonstrated progression to a large territorial infarct. Axial diffusion-weighted imaging (A) shows focal diffusion restriction in the left middle cerebral artery (inferior division) territory (arrowhead), along with multifocal infarcts (arrows), confirmed by true restricted diffusion on the corresponding ADC map (B). Additional DWI images (C and D) reveal small infarcts (arrows) in both hemispheres, involving the frontal, parietal, occipital, and temporal lobes, with associated mild mass effect. ADC: Apparent diffusion coefficient, DWI: Diffusion-weighted imaging

On day seven, the patient remained obtunded despite the absence of sedation. A follow-up MRI was obtained and demonstrated a territorial infarct in the left middle cerebral artery (inferior division) territory in addition to multifocal infarcts in both hemispheres involving the frontal, parietal, occipital, and temporal lobes, with associated mild mass effect and a 2 mm rightward midline shift (Figure 3). Clinically, the patient remained intubated and minimally responsive to painful stimuli. Her neurologic examination showed only spontaneous eye opening with minimal withdrawal to pain. Given her poor clinical condition, severe thrombocytopenia, and the unknown time of last known well, mechanical thrombectomy was not a viable option for her established stroke. Furthermore, data on the safety and efficacy of mechanical thrombectomy in patients with thrombotic thrombocytopenic purpura (TTP) and large vessel occlusion (LVO) are limited, and further research is warranted [11].

Despite aggressive treatment including daily plasma exchange, corticosteroids, rituximab, cyclophosphamide, and caplacizumab, ADAMTS13 activity and platelet count remained low, and ADAMTS13 inhibitor levels remained high, with only mild improvement. Clinically, the patient continued to deteriorate. Hematology and neurology consultants agreed that the prognosis for meaningful neurologic recovery was extremely limited. After discussion with the family, the decision was made to transition to comfort-focused care.

## Discussion

TTP is a rare but life-threatening thrombotic microangiopathy caused by a severe deficiency in ADAMTS13, a metalloprotease responsible for cleaving ultra-large von Willebrand factor (vWF) multimers. When ADAMTS13 activity is deficient, these multimers accumulate, leading to uncontrolled platelet aggregation and widespread formation of platelet-rich microthrombi in the microvasculature. The central nervous system is particularly vulnerable, and neurologic involvement, including encephalopathy, seizures, and ischemic stroke, occurs in the majority of patients [3].

This patient initially presented with altered mental status. Early MRI demonstrated multiple small, punctate infarcts scattered across bilateral deep gray matter structures, the cerebellum, and cortical regions, hallmarks of diffuse microvascular ischemia. These lesions, often described in TTP, are not confined to a single vascular territory and typically reflect widespread endothelial damage and microthrombi rather than embolic disease. In this context, such a pattern of multifocal punctate infarction should raise early suspicion for TTP, especially when paired with thrombocytopenia and hemolytic anemia [4].

Despite the early initiation of therapy, including daily plasma exchange (PLEX), high-dose corticosteroids, rituximab, cyclophosphamide, and caplacizumab [12-14], the patient’s condition deteriorated. She developed seizures, progressive encephalopathy, and ultimately a large left middle cerebral artery (MCA) territory infarction. This represented a significant shift from microvascular to large-vessel injury, suggesting either propagation of the thrombotic process [7,15]. One major factor contributing to the progression of cerebral ischemia in this case was the inability to initiate any form of antithrombotic therapy due to the patient’s profound thrombocytopenia. Antiplatelet agents and anticoagulants, which are standard components of secondary stroke prevention in ischemic stroke, are contraindicated in patients with platelet counts below 50,000/μL and especially so when counts fall below 10,000/μL [10], as they did in this case. Likewise, thrombolytic therapy was not an option at presentation due to absolute contraindication in severe thrombocytopenia. Thus, standard stroke-directed interventions were no longer applicable for halting progression to a large-vessel stroke, leaving the underlying TTP as the sole viable therapeutic target.

This case highlights a central therapeutic dilemma in TTP-related strokes: although the underlying pathophysiology involves widespread microvascular thrombosis, standard antithrombotic therapies such as antiplatelets, anticoagulants, or thrombolytics are typically contraindicated due to severe thrombocytopenia. As a result, clinicians must rely solely on disease-modifying therapies, such as plasma exchange (PLEX), immunosuppressants, and caplacizumab, to halt thrombotic progression. This places even greater urgency on the early initiation and optimization of these treatments. Further studies are needed to evaluate whether more aggressive or earlier use of advanced therapies can reduce the risk of stroke progression in TTP, particularly in refractory or fulminant cases.

Our case underscores the importance of recognizing TTP as a potential cause of stroke-like symptoms, especially when brain imaging reveals multifocal punctate infarcts and laboratory findings include thrombocytopenia and microangiopathic hemolysis. Clinicians must maintain a high index of suspicion and act swiftly to initiate disease-specific therapy to prevent irreversible organ damage. However, as this case demonstrates, neurologic complications may still progress despite optimal treatment, highlighting the limitations of current therapeutic strategies. The lack of safe and effective stroke prevention options in the setting of severe thrombocytopenia further complicates management and contributes to poor outcomes.

Ultimately, this patient's clinical trajectory from scattered microinfarcts to a catastrophic left middle cerebral artery (MCA) infarct with mass effect and encephalopathy illustrates the fulminant nature of refractory TTP. This progression occurred despite early diagnosis and the escalation of therapy, including the use of PLEX, corticosteroids, rituximab, cyclophosphamide, and caplacizumab. The case reinforces the need for early neurologic imaging, continuous reassessment, and timely escalation of therapy when initial responses are inadequate. Additionally, it raises an important question for future investigation: What is the safest and most effective platelet threshold for initiating antithrombotic therapy in patients with TTP-associated stroke? We believe further research is warranted to explore the lowest safe platelet threshold at which antithrombotic therapy can be initiated in patients with refractory and fulminant TTP in order to maximize therapeutic benefit while minimizing the risk of bleeding.

## Conclusions

Refractory TTP remains a critical clinical challenge with high mortality, particularly when complicated by recurrent cerebral infarctions and multiorgan dysfunction. This case underscores the importance of prompt diagnosis and early escalation of therapy, including plasma exchange, immunosuppressants, and caplacizumab. However, even with maximal treatment, irreversible neurologic injury may occur. Clinicians should maintain a high index of suspicion for TTP in patients presenting with neurologic symptoms, anemia, and thrombocytopenia, and recognize that despite optimal management, outcomes in refractory cases may remain poor. Studies are needed to determine if and when to start antithrombotic therapy to prevent stroke progression in TTP. Guidelines for managing stroke in TTP may also be warranted, highlighting the need for prospective research and guideline development.

## References

[1] Saha M, McDaniel JK, Zheng XL (2017). Thrombotic thrombocytopenic purpura: pathogenesis, diagnosis and potential novel therapeutics. J Thromb Haemost.

[2] Masias C, Cataland SR (2018). The role of ADAMTS13 testing in the diagnosis and management of thrombotic microangiopathies and thrombosis. Blood.

[3] Pishko AM, Li A, Cuker A (2025). Immune thrombotic thrombocytopenic purpura: a review. JAMA.

[4] Joly BS, Coppo P, Veyradier A (2017). Thrombotic thrombocytopenic purpura. Blood.

[5] Vasko R, Koziolek M, Füzesi L (2010). Fulminant plasmapheresis-refractory thrombotic microangiopathy associated with advanced gastric cancer. Ther Apher Dial.

[6] Zhu H, Liu JY (2022). Thrombotic thrombocytopenic purpura with neurological impairment: a review. Medicine (Baltimore).

[7] Sugarman R, Tufano AM, Liu JM (2018). Large vessel stroke as initial presentation of thrombotic thrombocytopenic purpura. BMJ Case Rep.

[8] Truma A, Mancini I, Agosti P (2024). Main features of ischemic stroke in patients with acute immune-mediated thrombotic thrombocytopenic purpura. Thromb Res.

[9] Zheng XL, Al-Housni Z, Cataland SR (2025). 2025 focused update of the 2020 ISTH guidelines for management of thrombotic thrombocytopenic purpura. J Thromb Haemost.

[10] Xu D, Zhou H, Zhang T (2024). Safety of antiplatelet therapy in noncardioembolic ischemic stroke with thrombocytopenia: the CASE II study. J Am Heart Assoc.

[11] Badugu P, Idowu M (2019). Atypical thrombotic thrombocytopenic purpura presenting as stroke. Case Rep Hematol.

[12] Scully M, Cataland SR, Peyvandi F (2019). Caplacizumab treatment for acquired thrombotic thrombocytopenic purpura. N Engl J Med.

[13] Sayani FA, Abrams CS (2015). How I treat refractory thrombotic thrombocytopenic purpura. Blood.

[14] Subhan M, Scully M (2022). Advances in the management of TTP. Blood Rev.

[15] Romozzi M, Vitali F, Marca GD (2023). Large vessel stroke and isolated thrombocytopenia as presenting features of thrombotic thrombocytopenic purpura. Neurol Sci.

